# A case report of early application of veno-arterial extracorporeal membrane oxygenation in amniotic fluid embolism

**DOI:** 10.1097/MD.0000000000027896

**Published:** 2021-11-19

**Authors:** Chen Ge, Junhang Liu, You Fu, Lijing Jia, Yinxiang Bai, Zhiwei Yang, Quansheng Du

**Affiliations:** a Department of Intensive Medicine, Hebei General Hospital, Shijiazhuang City, Hebei Province, P.R. China; b Department of Orthopaedics Surgery, Children's Hospital of Hebei, Shijiazhuang City, Hebei Province, P.R. China.

**Keywords:** amniotic fluid embolism, serial bedside ultrasound, venoarterial extracorporeal membrane oxygenation

## Abstract

**Rationale:**

Amniotic fluid embolism (AFE) is a rare obstetrical complication and is a leading cause of maternal death in developed countries. Despite the development of supportive therapeutic measures, the mortality rate remains high.

**Patient concerns:**

A 38-year-old nulliparous pregnant woman, who underwent in vitro fertilization-embryo transfer, was admitted for labor at 37 weeks’ gestation. Approximately 30 minutes after delivery of the placenta, the puerpera developed postpartum hemorrhage with uterine atony. Soon after, the patient experienced hypotension, repeated cardiac arrest, refectory hypoxia, and disseminated intravascular coagulopathy.

**Diagnosis:**

AFE is diagnosed clinically. The pregnant woman in this case fulfilled the diagnostic criteria for AFE: acute hypotension, cardiac arrest, acute hypoxia, and coagulation disorders within approximately 30 minutes after delivery of the placenta.

**Interventions:**

The patient was intubated, connected to a ventilator, and was administered a high dose of vasoactive drugs to maintain blood pressure and underwent an emergency hysterectomy. Considering the risk for recurrent cardiac arrest and severe refractory hypoxia, venoarterial extracorporeal membrane oxygenation was initiated and discontinued as soon as cardiac function was restored based on serial bedside ultrasound assessment.

**Outcomes:**

The patient stabilized on day 7 in the intensive care unit and was transferred to the obstetrics ward and, 1 week later, was discharged with no complications. Two months later, follow-up revealed that the patient was in good condition.

**Lesson:**

Serial bedside ultrasound was crucial for assessing cardiac function and optimal weaning. Timely application of venoarterial extracorporeal membrane oxygenation and weaning was significant to avoid the occurrence of complications and improve long-term outcomes.

## 1 Introduction

Amniotic fluid embolism (AFE) is a rare obstetrical complication, and a leading cause of maternal death in developed countries.^[1]^ The approximate incidence rate varies from 1.9/100,000 to 7.7/100,000 deliveries, with a mortality rate 19% to 86%.^[1–4]^ Currently, AFE is generally diagnosed clinically. It typically manifests as hypoxia, hypotension, and coagulopathy, which often rapidly progresses to cardiopulmonary collapse, cardiac arrest, and disseminated intravascular coagulopathy (DIC).^[5]^ The symptoms are transient and may improve within a short time; therefore, immediate and correct management focused on resuscitation and supportive care in the acute setting are particularly critical.^[6]^ However, a high mortality rate indicates that traditional treatment measures frequently do not provide sufficient support.^[3]^ Currently, there are a few case reports describing the use of extracorporeal membrane oxygenation (ECMO) in the treat of AFE.^[7,8]^ If a patient continues to exhibit life-threatening hemodynamic instability and refractory hypoxia, despite aggressive therapies, ECMO may be considered to provide cardiovascular support and oxygenate blood outside the body to temporarily replace the heart and lungs. Herein, we report a case of AFE involving a patient who experienced repeated cardiac arrest, refectory hypoxia, and DIC that was successfully treated with venoarterial extracorporeal membrane oxygenation (VA-ECMO).

## 2 Case report

A 38-year-old nulliparous pregnant woman, who underwent in vitro fertilization-embryo transfer was admitted for labor at 37 weeks’ gestation. One month before admission, hypertension was diagnosed, with a blood pressure (BP) of approximately 150/110 mm Hg. BP was controlled to approximately 130/90 mm Hg with administration of extended-release nifedipine tablets 10 mg once per day. The patient received regular prenatal care, and all laboratory investigations were normal. Three days after admission, induced delivery began with oxytocin when the patient experienced irregular contractions. The cervix was dilated to 10 cm 2 days after induction. Obstetrical forceps were used during delivery. A neonate was delivered with an Apgar score of 6 at 1 minute. The neonate was intubated immediately after birth and transferred to the neonatal intensive care unit.

**Figure 1:**
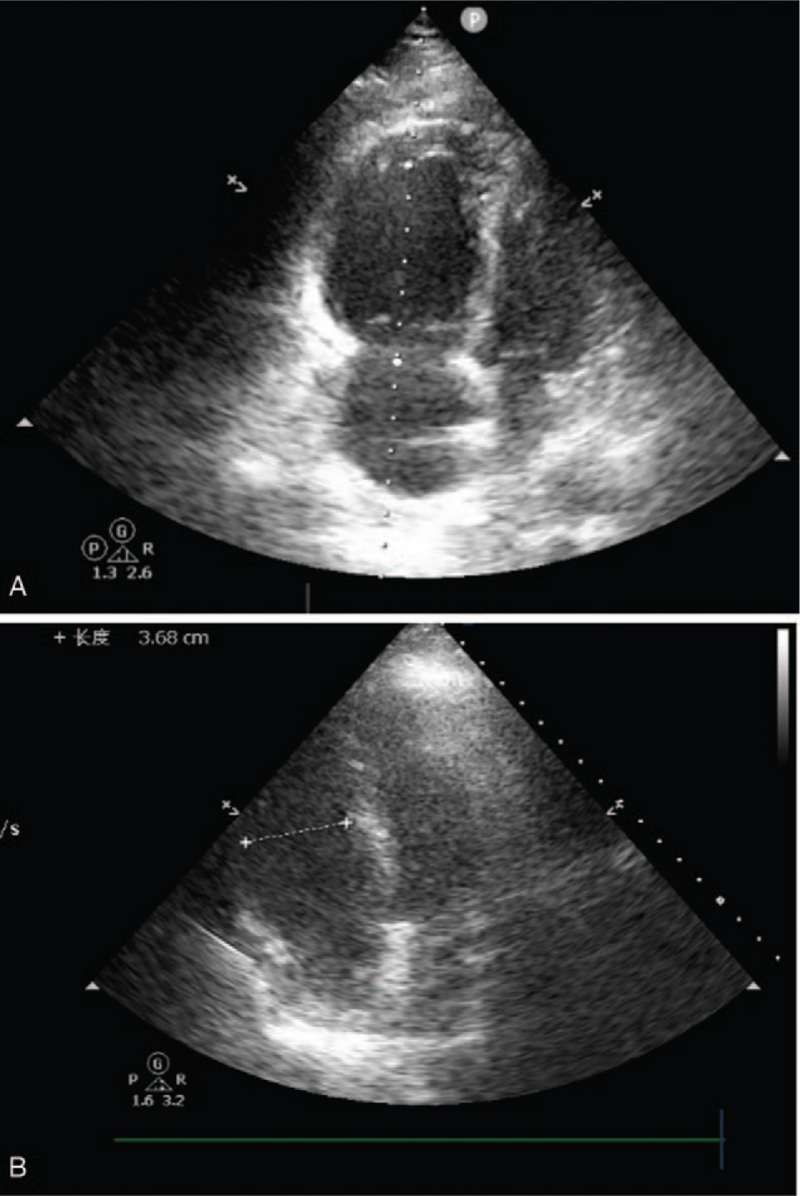
Bedside cardiac ultrasound of the patient before and after carrying out ECMO. (A) Bedside cardiac ultrasound showed right heart expansion (RV 44 mm) before carrying out ECMO. (B) Bedside cardiac ultrasound showed right heart size dropped to normal (RV 36 mm) after carrying out ECMO. ECMO = extracorporeal membrane oxygenation.

**Figure 2:**
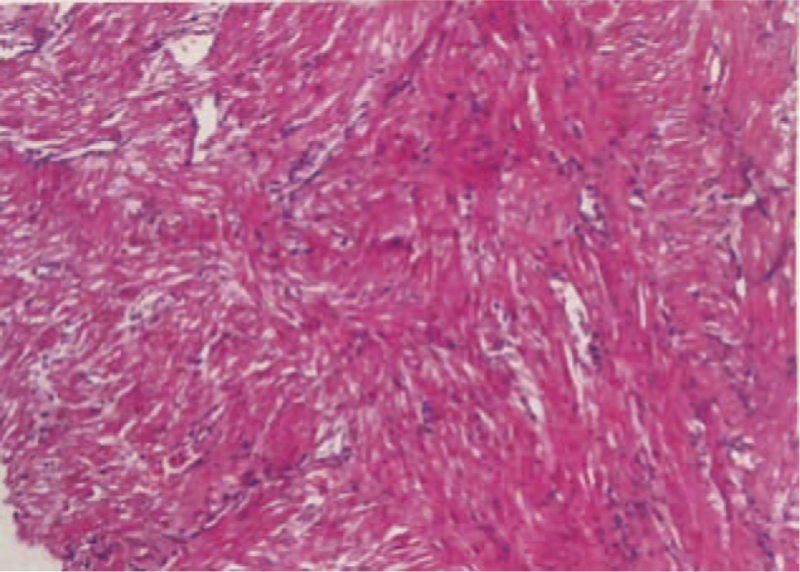
Pathological morphology of uterine by HE staining at 40× magnifications. Blood vessels of uterine showed meconium and keratin-like material.

Approximately 30 minutes after delivery of the placenta, puerpera developed postpartum hemorrhage with uterine atony. Soon after, the mother became dysphoric, hypoxic, and hypotensive, with the following vital signs: non-invasive BP, 70/43 mm Hg; heart rate, 123 beats/min; and oxygen saturation, 43%. The patient was promptly intubated and connected to a ventilator supplying 100% oxygen. A central venous line was placed while large amounts of fluid, including blood products and vasoactive drugs, were infused to maintain BP. Radial artery catheters were placed to monitor the BP. Laboratory investigations revealed a prolonged prothrombin and activated partial thromboplastin times, elevated fibrinogen level, platelet count, and D-dimer levels, suggestive of DIC. Arterial blood gas analysis revealed severe hypoxia, metabolic acidosis, and severe lactic acidosis. The obstetrician placed a Cook balloon to stop uterine bleeding. However, uterine hemorrhage continued, and 2 consecutive episodes of cardiac arrest developed (90 seconds for the first and 3 minutes for the second). Cardiopulmonary resuscitation was performed, and epinephrine (1 mg) was administered. Active movements were observed after the return of spontaneous circulation. With persistent uterine bleeding, the obstetrician decided to perform a total hysterectomy. For refractory hypoxia (oxygenation index < 50), hypotension (norepinephrine 2 μg/kg/min, dopamine 5 μg/kg/min) and DIC, it was suspected that the patient experienced AFE. Bedside ultrasound revealed right heart expansion (right atrium, 42 mm; right ventricle, 44 mm) and pulmonary arterial hypertension (pulmonary arterial systolic pressure 60 mm Hg); however, left ventricular systolic function did not decrease (ejection fraction 68%) (Fig. 1A). Both ultrasound and uterine pathological findings (Fig. 2) reported later confirmed AFE. In consideration of the risk for recurrent cardiac arrest and severe refractory hypoxia, VA-ECMO was initiated 90 minutes after the onset of hypotension and hypoxia. Hydrocortisone (200 mg) and papaverine (90 mg) were administered to improve pulmonary hypertension at the onset of hypotension and hypoxia-suspected AFE. Both hysterectomy and ECMO were performed in the delivery room due to the hemodynamic instability of the puerpera. Obstetricians, intensive care unit physicians, cardiologists, and anesthesiologists participated in the entire rescue process. Approximately 3500 mL of blood was lost, and 42 units of red blood cells, 3000 mL of fresh frozen plasma, 20 units of platelets, and 50 units of cryoprecipitate was transfused during the rescue process.

With the support of ECMO (heparin-free, flow rate 2.68 L/min, 2000 revolutions/min, sweep gas rate 3 L/min, oxygen concentration 60%), the patient's circulation stabilized (norepinephrine 1 μg/kg/min, dopamine 5 μg/kg/min) and oxygenation improved (oxygenation index, 300). The patient was transferred to the intensive care unit. The patient's heart was assessed approximately every 4 hour using serial bedside ultrasound. Right heart size and pulmonary arterial hypertension dropped to normal after ECMO for nearly 20 hours (Fig. 1B). Vasoactive drugs were then discontinued. As circulation and oxygenation improved, weaning from ECMO was attempted. Before weaning, however, it was decided to evaluate cardiac function by lowering the ECMO parameters as much as possible (heparin-free, flow rate, 1.5 L/min, sweep gas rate, 3 L/min; oxygen concentration, 40%). Right heart size and pulmonary arterial hypertension were normal. Therefore, the patient was decannulated, with ECMO support for nearly 20 hours.

**Figure 3:**
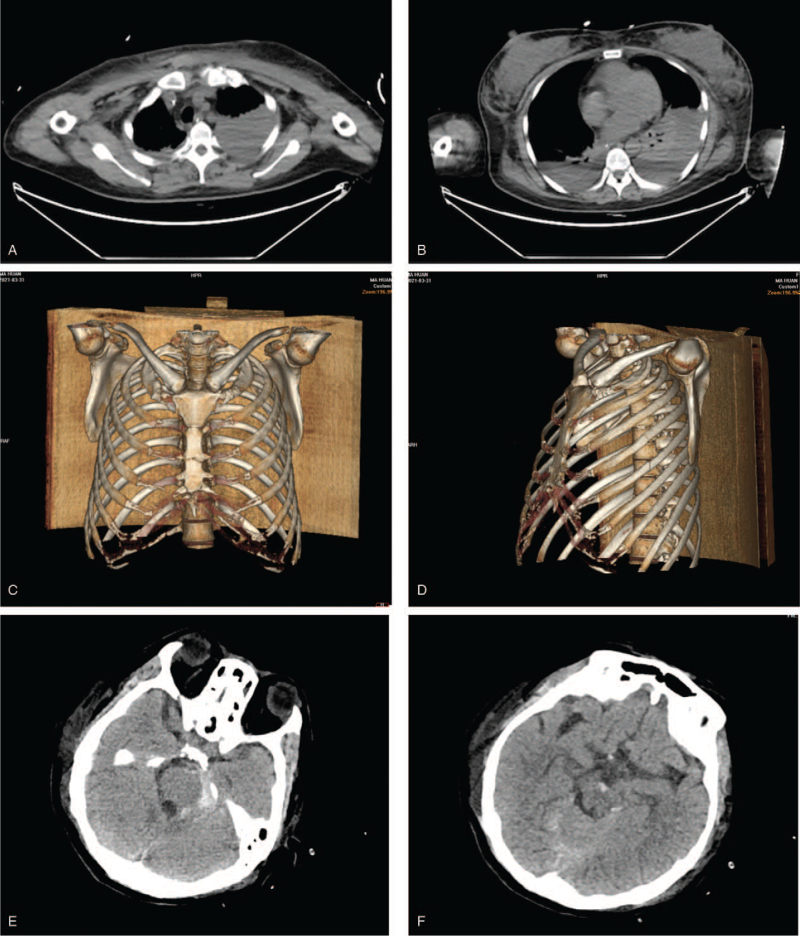
CT scans of the chest and brain in the ICU on day 1. (A) & (B) CT scans of the chest showed a large left pleural effusion. (C) & (D) CT scans of the chest showed fractures of the sternum and left ribs (4th and 5th ribs). (E) & (F) CT scans of brain showed subarachnoid hemorrhage. CT = computed tomography, ICU = intensive care unit.

On days 1 and 2, blood clotting indexes improved with blood transfusion; however, hemoglobin level progressively decreased. A large left pleural effusion was found, in addition to fractures of the sternum and left ribs (4th and 5th ribs), and subarachnoid hemorrhage by computed tomography examination (Fig. 3). Thorax puncture and catheterization were performed to drain the bloody pleural effusion. Blood transfusion was performed to improve clotting indexes and platelet levels.

**Figure 4:**
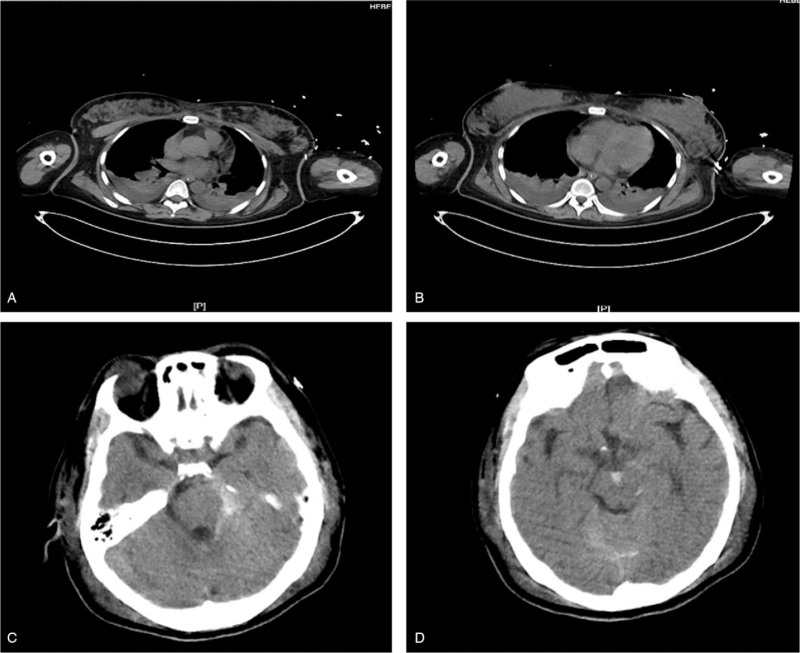
CT scans of the chest and brain in the ICU on day 5. (A) & (B) CT scans of the chest showed the left pleural effusion was significantly reduced. (C) & (D) CT scans of brain showed the area of subarachnoid hemorrhage was not enlarged. CT = computed tomography, ICU = intensive care unit.

On day 3, hemoglobin level stabilized without blood transfusion, and no pleural effusion was observed. The patient was placed under deep sedation due to a subarachnoid hemorrhage. Maintenance of negative balance of capacity was used to improve oxygenation. Computed tomography revealed that the area of subarachnoid hemorrhage was not enlarged, and the left pleural effusion was significantly reduced on day 5 (Fig. 4). Therefore, sedative was reduced to observe the patient's level of consciousness. Weaning from mechanical ventilation and extubation was performed on day 6. On day 7, the patient was transferred to the obstetrics ward. One week later, the patient was discharged from the obstetrics ward. Two months later, follow-up revealed that the patient was in good condition with no complications. And the patient has consented to publish this case report.

## 3 Discussion

Presently, risk factors for AFE include advanced age, cesarean delivery, medical induction of labor, placenta previa, and accrete.^[1,9]^ Other risk factors that may be associated with AFE include the use of oxytocin, cervical lacerations, uterine rupture, eclampsia, polyhydramnios, and multiple birth.^[10–12]^ With amniotic fluid or fetal materials entering the maternal circulation, the pulmonary vessels experience spasms. Hypoxia and decreased left ventricular function occur as a result of pulmonary hypertension. Finally, the patient experienced cardiopulmonary collapse, cardiac arrest, uterine dystonia, and DIC. AFE often first presents with bleeding due to consumptive coagulopathy.

AFE was initially believed to be caused by emboli in the pulmonary vasculature. However, this opinion has been overturned because pulmonary embolism is rarely observed.^[6,13]^ A new hypothesis was proposed, in which AFE is believed to be highly similar to anaphylaxis.^[14]^ This appears to be more appropriate as an anaphylactic syndrome of pregnancy.^[15]^ Therefore, the destructive symptoms are transient and recoverable within a short time, even a few hours. AFE is clinically diagnosed and pregnant women must exhibit the following 5 clinical characteristics: acute hypotension or cardiac arrest; acute hypoxia; coagulation disorder(s) in the absence of other explanations; symptoms occurring during labor, cesarean delivery, curettage, or within 30 minutes after delivery of the placenta; and signs and symptoms described above cannot be explained by other diseases.^[16]^ The patient in our case met these diagnostic criteria and pathological analysis confirmed the diagnosis.

ECMO can temporarily replace cardiopulmonary function to ensure sufficient oxygen supply and reduce cardiac oxygen consumption in patients experiencing anaphylaxis. A few previous case reports have described the use of VA-ECMO to temporarily replace a dysfunctional cardiopulmonary system to treat cardiac arrest after AFE.^[17,18]^ VA-ECMO has been proposed to replace non-resuscitated hearts after cardiac arrest.^[19]^ In our case, ultrasound clearly demonstrated elevated pulmonary arterial pressure and enlargement of the right heart induced repeated cardiac arrest, which directly led to the decision to initiate VA-ECMO. The venoarterial approach should be preferred to manage cardiac failure, while the veno-venous approach should be limited to pure respiratory failure.^[20]^ The extracorporeal life support registry has reported a 57% mortality rate in adults who received ECMO for cardiogenic shock, with worse outcomes related to prolonged cannulation.^[21]^ In this case, we performed VA-ECMO 1 hour after the first cardiac arrest. Early and timely implementation of VA-ECMO can reduce cardiac load and ensure sufficient oxygen supply until transient acute anaphylaxis is over. Early application of ECMO is also critical to protect brain function by ensuring oxygen delivery to the brain. A summary of several case reports of AFE is presented in Table 1.^[7,22–27]^

**Table 1:** Seven case reports of amniotic fluid embolism applied with ECMO.

Reference No.	Age (yrs)	When ECMO was started	Type of ECMO	When ECMO was weaned	Prognosis
7	34	Hemodynamic instability, heart failure	VA-ECMO, IABP	Restoration of hemodynamic stability, recovery of heart function, relative stable vital signs	The patient was discharged after 24 days of hospitalization with no complication.
22	33	Chest tightness, dyspnea, loss of consciousness, asystole	VA-ECMO	Relative stable vital signs	After a 2-week stay in the ICU for care, the patient was returned to the ward with left-side muscle power weakness.
23	35	Unresponsive, cyanotic, bradycardic, repeated cardiac arrest, 2.5 hours of CPR	VA-ECMO	Improved cardiac function	The patient made a full cognitive recovery with a cerebral performance score of 1 and mild right hand motor weakness after a 13-day hospitalization.
24	37	Severe postpartum, hemorrhage, hypoxic respiratory failure, cyanotic, hypotension	VV-ECMO	Controlled bleeding and improved vital signs	The patient was successfully discharged home with minimal sequelae.
25	42	Unconscious, hypotension, hypoxia, cardiacvascular shock, cardiac arrest	VA-ECMO	Decreased thrombus load, resolution of RV dilatation, progressive improvement in contractility	The patient regained baseline neurological function and was discharged to a rehabilitation center after a 25-day hospitalization.
26	36	Postpartum hemorrhage, hemodynamic instability, neurological deterioration, 3 consecutive episodes of cardiac arrest, stunned myocardium revealed by transthoracic echocardiography	VA-ECMO	Restoration of hemodynamic stability, improved hypoxemia	Two months after delivery, mother and child were both alive and well. Transthoracic echocardiography showed recovery of normal heart function.
27	39	Generalized tonic-clonic seizures, semicoma, DIC, severe lactic acidosis, bilateral pleural effusion showed by chest X-ray, hypoxemia, heart failure, hypotension	VA-ECMO	Relative normal vital signs	41 days after delivery, the patient remains hospitalized and is receiving rehabilitation with nerve injury complications in the right lower extremity where ECMO was inserted.

CPR = cardiopulmonary resuscitation, DIC = disseminated intravascular coagulopathy, ECMO = extracorporeal membrane oxygenation, IABP = intra-aortic balloon counterpulsation, ICU = intensive care unit, RV = right ventricular, VA-ECMO = venoarterial extracorporeal membrane oxygenation, VV-ECMO = Veno-Venous extracorporeal membrane oxygenation.

However, ECMO may be associated with complications, including bleeding caused by anticoagulation, hemolysis, renal kidney injury, infection, and limb ischemia.^[21]^ To avoid these complications, timely weaning from ECMO is as important as early initiation. Patients should be weaned off ECMO as soon as possible. A few case reports have suggested that indications for ECMO weaning include improved primary disease, adequate cardiac function, and relatively stable vital signs.^[13–16]^ In our case, we observed that the right heart size and pulmonary arterial hypertension dropped to normal, circulation and oxygenation improved; as such, we attempted to wean the patient off ECMO. Previous case reports have indicated that ECMO support time is probably >24 hours, even up to 150 hours, although complications may occur.^[8,17,28]^ In the present case, our patient was successfully weaned from ECMO after <20 hours, with virtually no complications, which is different from previous case reports. This benefit may be attributed to early and timely initiation of ECMO, which was initiated 1 hour after the first cardiac arrest. Assessment of cardiopulmonary function is also particularly important. This, however, requires the evaluation of cardiopulmonary function in real time. In the present case, we assessed the status of the patient's heart every 4 hour using serial bedside ultrasound and reduced the ECMO flow rate when the patient's cardiac function improved, and cardiac function was reassessed to be in good condition. The patient was urgently decannulated to avoid complications. The patient was supported by ECMO for 20 hours using a heparin-free approach, given the presence of coagulation disorders and bleeding.

## 4 Conclusion

Randomized controlled trials investigating AFE treatment with ECMO, including the timing of initiation of ECMO, remain lacking. In the present case, early VA-ECMO support for acute cardiopulmonary dysfunction was found to be beneficial. There are challenges to balancing ECMO with coagulation dysfunction. For these patients, a heparin-free ECMO strategy may be considered. Serial bedside ultrasound is crucial for assessing cardiac function and optimal weaning. Timely and correct weaning was significant to avoid the occurrence of complications and improve long-term outcomes.

## Author contributions

**Project administration:** Quansheng Du.

**Resources:** Yinxiang Bai, Quansheng Du.

**Supervision:** Lijing Jia, Quansheng Du.

**Writing – original draft:** Chen Ge, Junhang Liu, You Fu, Zhiwei Yang.

**Writing – review & editing:** Chen Ge, Lijing Jia, Quansheng Du.
